# Barley *Hv CIRCADIAN CLOCK ASSOCIATED 1* and *Hv PHOTOPERIOD H1* Are Circadian Regulators That Can Affect Circadian Rhythms in Arabidopsis

**DOI:** 10.1371/journal.pone.0127449

**Published:** 2015-06-15

**Authors:** Jelena Kusakina, Zoe Rutterford, Sean Cotter, María C. Martí, David A. Laurie, Andy J. Greenland, Anthony Hall, Alex A. R. Webb

**Affiliations:** 1 Institute of Integrative Biology, University of Liverpool, Crown Street, Liverpool, United Kingdom; 2 Department of Plant Sciences, University of Cambridge, Downing Street, Cambridge, United Kingdom; 3 National Institute of Agricultural Botany, Cambridge, United Kingdom; 4 John Innes Centre, Coney Lane, Norwich, United Kingdom; Ohio State University, UNITED STATES

## Abstract

Circadian clocks regulate many aspects of plant physiology and development that contribute to essential agronomic traits. Circadian clocks contain transcriptional feedback loops that are thought to generate circadian timing. There is considerable similarity in the genes that comprise the transcriptional and translational feedback loops of the circadian clock in the plant Kingdom. Functional characterisation of circadian clock genes has been restricted to a few model species. Here we provide a functional characterisation of the *Hordeum vulgare* (barley) circadian clock genes *Hv CIRCADIAN CLOCK ASSOCIATED 1 *(*HvCCA1*) and *Hv PHOTOPERIODH1*, which are respectively most similar to *Arabidopsis thaliana CIRCADIAN CLOCK ASSOCIATED 1 (AtCCA1)* and *PSEUDO RESPONSE REGULATOR 7 (AtPRR7)*. This provides insight into the circadian regulation of one of the major crop species of Northern Europe. Through a combination of physiological assays of circadian rhythms in barley and heterologous expression in wild type and mutant strains of *A*. *thaliana* we demonstrate that *HvCCA1* has a conserved function to *AtCCA1*. We find that *Hv PHOTOPERIOD H1* has *AtPRR7*-like functionality in *A*. *thaliana* and that the effects of the *Hv photoperiod h1* mutation on photoperiodism and circadian rhythms are genetically separable.

## Introduction

Circadian clocks are timing mechanisms that are an adaptation to the Earth’s rotation. These circadian clocks are entrained to the day/night cycle by sensing environmental cues such as light and temperature and act as master regulators to synchronise biological events to specific times of the day [1]. In plants, the circadian clock has been shown to control approximately one third of the genome and it regulates a wide range of key processes such as hypocotyl elongation, leaf movement, stomatal opening and flowering [1]. Furthermore, accurate and robust circadian function enhances yield, water use efficiency and overall plant performance [2,3].

While the molecular components of the clock are not conserved across taxonomic groups, most circadian oscillators comprise a number of coupled transcription/translation feedback loops [4]. In the model dicotyledonous plant Arabidopsis, the central clock is proposed to consist of several interlocking loops [5]. A loop of morning active genes is formed by the partially redundant MYB-transcription factors *AtCCA1* (*CIRCADIAN CLOCK ASSOCIATED 1*) and *AtLHY* (*LATE ELONGATED HYPOCOTYL*) and two members of the pseudo-response regulators family, *AtPRR7* (*PSEUDO-RESPONSE REGULATOR* 7), and *AtPRR9 (PSEUDO-RESPONSE REGULATOR 9)*. *AtCCA1/LHY* activate *AtPRR7/9* and in turn *AtPRR7/9* repress *AtCCA1/LHY*. A loop of evening-active genes is formed by *AtGI* (*GIGANTEA*) and *AtPPR1/TOC1* (*PSEUDO-RESPONSE REGULATOR 1/TIMING OF CAB EXPRESSION)* which both negatively regulate each other. The loops are coupled by the repressive effects of *AtCCA1/LHY* on *AtTOC1* and *AtTOC1* on *AtCCA1/LHY* [6,7]. Another loop of mutual repression exists between the morning loop and an “evening complex” of *AtELF3*, *AtELF4* and *LUX* (*EARLY FLOWERING 3*, *4* and *LUXARRHYTHMIO*) through recruitment of the transcriptional repressor LUX by ELF3 to the *PRR9* promoter [8].

Monocot circadian clocks appear to be comprised of a similar structure. For example, in *Lemna gibba*, a floating monocotyledon plant, orthologues for *AtCCA1*, *AtLHY*, *AtGI*, *AtELF3* and several genes similar to the *AtPRR* family have been identified and their function in the *Lemna* circadian clock characterised [9]. In rice, another model monocot plant, several orthologues of central clock genes have also been identified, including *OsGI*, *OsZTL (ZEITLUPE)*, *OsCCA1/LHY* and members of the *OsPRR* family [10]. These components are under circadian control with similar expression patterns reported for homologous clock genes in Arabidopsis.

Most monocots contain only a single orthologues of *AtCCA1/LHY*. For example, rice has a single orthologues of *AtCCA1/LHY* (*OsLHY*) which functions in a similar manner to the Arabidopsis, acting as a repressor of the rice *AtTOC1* orthologues [11]. However, OsLHY does not homodimerize due to the loss of a key phosphorylation site, which demonstrates divergence between the rice and Arabidopsis clocks [11].

Barley contains orthologues of Arabidopsis circadian clock genes, with transcripts that are rhythmic suggesting that *HvCCA1*, *HvGI* and *HvPRR1* have a functions similar to their Arabidopsis counterparts [12]. For some of the *PRR* homologues it has proven difficult to determine which barley gene is ortholgous to Arabidopsis members of the family and therefore the designation reflects the uncertainty. *HvPRR37*, *HvPRR73*, describe two genes both of which are ortholougues to either *AtPRR3* or *AtPRR7*, and *HvPRR59* and *HvPRR95* are orthologus to either *AtPRR5* or *AtPRR9* [12]. Other circadian clock genes are thought to be important for agronomic traits such as *HvLUX1* and *HvELF3* underlying the *EARLY MATURITY 10* [13] and *8* [14] loci respectively.

*HvPRR37* is also known as *Hv PHOTOPERIOD H 1* (*HvPpd-H1*), which controls sensitivity to photoperiod. The *Ppd-H1* region contains a single *PRR* gene originally thought to be most similar to *AtPRR7* [15]. Landraces from south-west Asia, southern Europe, and the Mediterranean basin have the *Ppd-H1* allele that confers early flowering in long days of the spring. Whereas the photoperiodic-insensitive *ppd-H1* allele is present in landraces from central and northern Europe (this is known as spring barley, because it is planted in the spring and flowers at the end of summer). The reduced response to photoperiod of *ppd-H1* allows spring-sown plants to extend the period of vegetative growth and accumulate additional biomass that supports higher yields [15]. The late-flowering *ppd-H1* allele is recessive, suggesting that reduced response results from a mutation that impairs gene function [15]. The modern variants of barley used to map *Ppd-H1* were Igri which contains the photoperiod-sensitive allele (*Ppd-H1*) and Triumph which is late flowering (*ppd-H1*). The central role of *HvPpd-H1* (and *TaPpd-*D1a) in flowering is well characterised, but it is not known whether *HvPpd-H1* is a circadian clock gene in barley, or whether it is functionally equivalent to *AtPRR7* or *AtPRR3* because natural variation at the *HvPpd1-H1* has no effect on circadian rhythms of circadian clock gene transcripts but does affect the timing of expression of genes associated with flowering time [12, 14].

Barley represents a challenging system for studying circadian behaviour and it does not have the wide resource tools for molecular genetics developed for the model Arabidopsis, therefore we have used a heterologous approach to examine barley clock gene function by misexpression in Arabidopsis. We provide evidence that the barley circadian clock genes have conserved functions with their counterparts in Arabidopsis. We demonstrate that *Hv CCA1* has *AtCCA1-*like activity and *HvPpd-H1* has *AtPRR7-*like functionality in Arabidopsis. We suggest that the effects of *HvPpd-H1* on photoperiodism and circadian rhythms are separable. Our data suggests that flowering time in crops can be manipulated by appropriate breeding strategies without compromising circadian clock function.

## Methods

### Cloning of *HvCCA1* into Arabidopsis

The entire HvCCA1 transcript was amplified using KOD TAQ polymerase (Novagen). HvCCA1 was cloned into GATEWAY pCR8/GW/TOPO entry vector using pCR8/GW/TOPO TA cloning kit (Invitrogen) following the manufacturer’s instructions. HvCCA1 was then subcloned into the binary plazmid PMDC32 using the Gateway LR recombination reaction (Invitrogen). The binary plasmid PMDC32 confers hygromycin resistance in plants and contains a double 35S cauliflower mosaic virus promoter for constitutive expression of the inserted gene [16]. The CaMV35S:HvCCA1 was introduced into GV3101 Agrobacterium tumefaciens and subsequently into Arabidopsis thaliana Ws-2 via the floral dip method [17]. Out of 21 primary transformants, nine were identified as homozygous lines carrying an insert at a single locus. HvCCA1 overexpression was confirmed by RT-PCR (S1 Fig). Homozygous lines were used for all experiments.

### RNA extraction and transcript analysis

For circadian experiments seedlings were grown at 22°C in 12 h:12 h L:D for 7 days before being transferred to continuous light for 54 h. Samples were taken every 6 h until 72 h. Plant tissue was disrupted in the TissueLyser (QIAGEN) and total RNA was extracted using RNeasy plant kit (Qiagen). cDNA was synthesised from 0.5 μg of template RNA using the QuantiTect Reverse Transcription kit (Qiagen). For quantitative PCR, transcript abundance was measured using Power SYBR Green PCR master mix (Applied Biosystems, USA) in the Applied Biosystems 7500 Fast Real-Time PCR System. Transcript abundance of *AtCCA1*, *AtLHY* and *AtTOC1* was normalised to *UBIQUITIN10* (UBQ). The primers for q-PCR reactions are presented in S1 Table. All reactions were carried out in triplicate. Normalization and transcript abundance calculations were automated and performed using the Applied Biosystems 7500 Fast Real-Time PCR System software.

### Non-homologous Complementation of Arabidopsis *PRR* Mutants

Arabidopsis *PRR7* T-DNA insertion mutant (*prr7-11*) was complemented with *HvPpd-H1* and *Hvppd-H1* to test for similarity of function between *AtPRR7* and *HvPpd-H1*. pENTRPpdH1 (Igri) cDNA and of pENTRppdH1 (Triumph) were generated, each containing a fragment comprising the coding region of the *HvPpdH1* or *HvppdH1* cDNA from Cultivar “Igri” or “Triumph” respectively, cloned directionally into a pENTR-D plasmid (Invitrogen, UK). The genomic *AtPRR7* (AT5G02810) promoter (p) sequence was used to drive the *HvPpd-H1* cDNA sequences extending from -871 to +1 bases where the first nucleotide of the inferred ATG initiation codon. The promoter was amplified (S1 Table) from *pAtPRR7*:*LUC*, a luciferase binary vector supplied by C. Robertson McClung, originally derived from pZPomegaLUCplus [18] by replacement of the gentamicin resistance cassette with the *BASTA* resistance gene from p35SBarn [19]. The resulting vector, pZPBAR, was then made Gateway compatible (Invitrogen, Carlsbad, CA) by inserting the PCR-amplified attR-flanked destination cassette from pK7WG2D [20] at the *Bam*HI and *Hind*III sites upstream of *LUC* to create pZPBAR-DONR. The *AtPRR7* promoter was inserted into pZPBAR-DONR. The binary vector used was pBGW, supplied by the Functional Genomics unit of the Department of Plant Systems Biology (VIB-Ghent University). The 894 bp *pAtPRR7* product and pENTR-D Topo Ppd-H1 and pENTR-D Topo ppd-H1 recipient plasmids were digested with SacII restriction enzyme. The digested pENTR-D Topo PpdH1 and pENTR-D Topo ppdH1 samples were dephosphorylated by adding Shrimp Alkaline Phosphatase (SAP) buffer (Fermentas, UK),. The *pAtPRR7* insert DNA and pENTR-D Topo PpdH1 / ppdH1 vector backbones were mixed in a ratio of 3:1 (insert:backbone) with ligase (New England BioLabs, UK) and incubated at 4°C overnight. pENTR-D Topo PpdH1 or pENTR-D Topo ppdH1 with the AtPRR7 promoter were introduced into chemically competent DH5α E. coli cells (Invitrogen, UK). Colony PCR was used to identify bacteria carrying the pENTR-D Topo PpdH1 or pENTR-D Topo ppdH1 with the AtPRR7 promoter in the correct orientation. Plasmid DNA was isolated using Promega miniprep Wizard kits (Promega, UK). The insertion and orientation of *AtPRR7* into the pENTR-D Topo backbones was verified again by restriction digestion alongside the parent plasmids using SacI and SacII. Plasmids carrying the *AtPRR7* promoter in the correct insertion were fully sequenced through the AtPRR7 promoter and the Igri or Triumph coding regions. *A*. *tumefasciens* strain GV3101 was transformed with *pBGW-PRR7*:*PpdH1* or *pBGW-PRR7*:*ppdH1* and then used to transform Arabidopsis by floral dip [17]. Positive transformants were selected by spray with Finale glufosinate ammonium (0.31% v/v; Bayer, Germany). For physiological assays the F2 transgenic plants were used.

To ensure functionality of the promoter construct tissue was collected from 12-day-old seedlings grown in 12 h light–12 h dark cycles (100 μmol m−2 s−1) six h after dawn. Total RNA was extracted from frozen tissue of three biological replicates of at least five pooled plants each, using the RNeasy Plant Mini Kit (QIAGEN) and RNase-Free DNase on-column treatment (QIAGEN). cDNA was synthesized from 500 ng RNA with the RevertAid First Strand cDNA Synthesis Kit (Thermo Scientific) using oligo(dT) primers. The gene-specific products were amplified using the Rotor-Gene SYBR Green PCR Kit on a Rotor-Gene 6000 Real-Time PCR machine (QIAGEN). The primer sequences were: PP2a-F, 5′-TAACGTGGCCAAAATGATGC-3′; PP2a-R, 5′-GTTCTCCACAACCGCTTGGT-3′ and Hvppd-H1-F, 5′-GATGGATTCAAAGGCAAGGA-3′; Hvppd-H1-R, 5′-GAACAATTGGCTCCTCCAAA-3′ [12]. Relative transcript levels were determined by incorporating PCR efficiencies [21].

### Circadian rhythms of leaf movement, delayed fluorescence and analysis of hypocotyl elongation and flowering time in Arabidopsis

For leaf movement, delayed fluorescence and hypocotyl elongation experiments Arabidopsis seed was surface-sterilized in 70% ethanol for 1 min, followed by 50% (w/v) bleach for 10 min and 3 rinses with distilled water. Seeds were resuspended in 0.15% (w/v) agar and stratified at 4°C for 3 days prior to sowing on Murashige and Skoog medium 1.5% (w/v) agar plates. For leaf movement, ten day old seedlings entrained to 12h light 12 h dark cycles were moved to Sanyo MLR350 plant growth chambers and imaged under constant light at 22°C [22].

Delayed chlorophyll fluorescence was imaged in a Sanyo MIR-553 cooled incubator (Sanyo Gallenkamp, UK) using an ORCA-II-BT 1024 16-bit camera (Hamamatsu Photonics, Japan) cooled to -80°C [20]. Images from leaf movement and delayed fluorescence were processed using Metamorph 6.0 image-analysis software (Molecular Devices, UK). Period estimates and relative amplitude errors (RAE) were calculated in BRASS (available from http://www.amillar.org/downloads.html) by running fast Fourier transformed non-linear least-square analysis [23]. RAE is a measure of rhythm robustness that ranges from 0 (a perfect fit to the wave) to 1 (no fit).

Hypocotyl length was measured in ten day old seedlings grown at 22°C in 12h light 12 h dark of 100 μmol m^-2^ s^-1^. Seedlings were harvested and laid flat on agar plates for imaging. Images of Arabidopsis seedlings were analysed in Metamorph 6.0. Student’s *t*-test was used to determine statistical significance between the controls and mutants.

Flowering time assays were performed on Arabidopsis seedlings grown in a controlled environment under 16h light 8 h dark at 22°C. The flowering time was expressed as a number of days and rosette leaves when a bolt of 0.5 cm long was observed.

### Infra-Red Gas Exchange Analyser (IRGA) Assay of Circadian Rhythms in Arabidopsis and barley

Circadian rhythms of gas exchange in Arabidopsis plants was measured as described in [24]. For measurement of circadian gas exchange in individual barley plants an IRGA system based on the portable LI-COR LI-6400XT (LI-COR, USA) was developed. Humid CO_2_ free air was obtained by pumping atmospheric air through two 4.5 cm x 30 cm cylinders in series filled with soda lime (Fisher Scientific, UK) and a flask of water. Relative humidity was limited to a maximum of 85% through the control of the IRGA. CO_2_ (BOC, UK) was provided at between 180 and 220 PSIG and a flow of 500 μmol s-^1^. The [CO_2_] in the chamber was controlled at 380 PPM. A 6 cm^2^ area of mature barley (4–8 weeks old) leaf was clamped in the cuvette with a clear glass screen and therefore received illumination from the cabinet equivalent to that of the rest of the plant. Gaseous exchange was recorded every 10 min.

## Results

### Over-expression of *HvCCA1* causes arrhythmia of the Arabidopsis circadian clock

**Fig 1:**
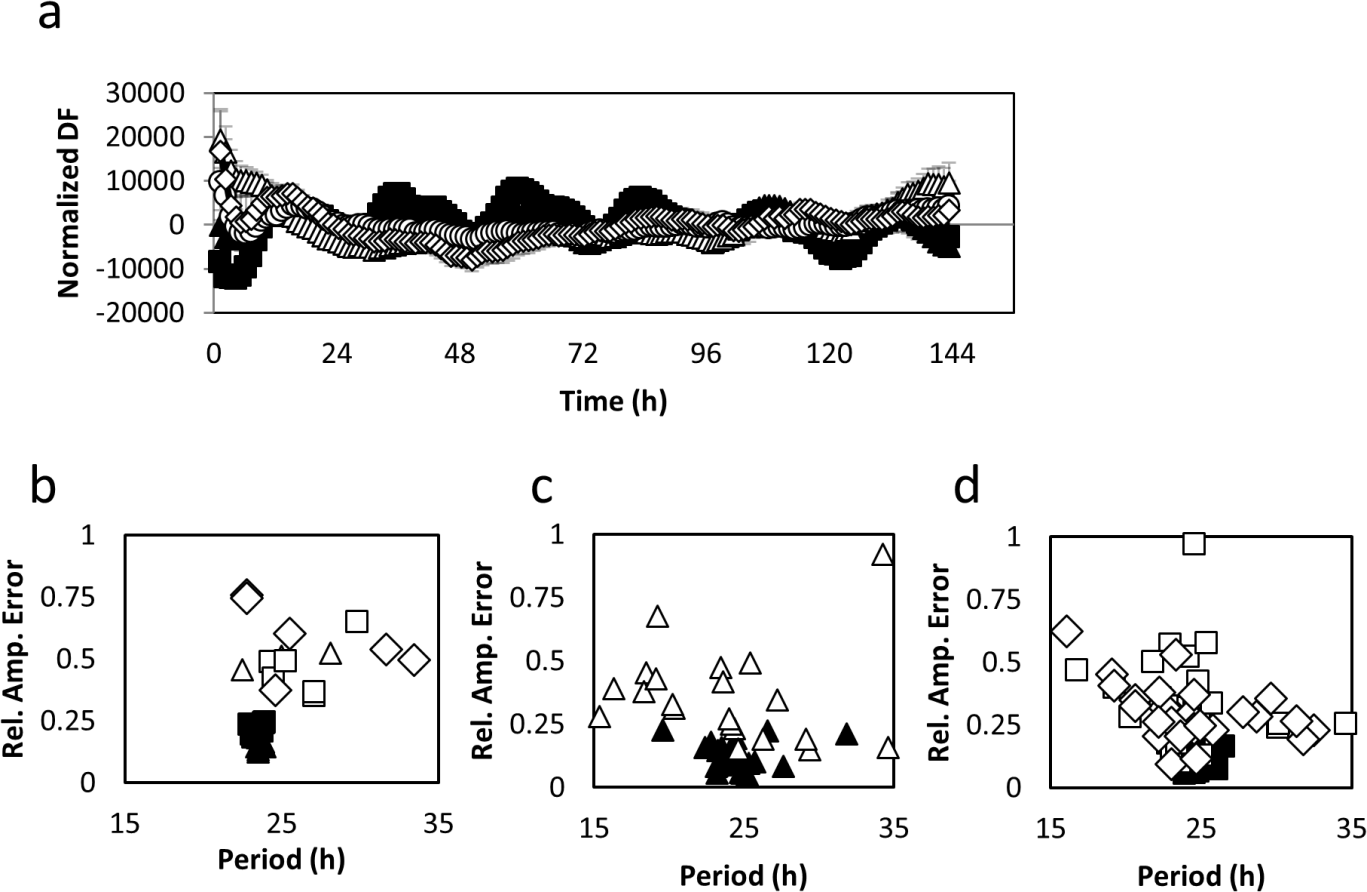
Over expression of *HvCCA1* causes circadian arrhythmia in Arabidopsis. Normalised delayed chlorophyll fluorescence (a) and period estimates vs R.A.E (b) for Ws-2, Col-0, *AtCCA1*-ox (*AtCCA1*-ox 038) and two independent *HvCCA1-*ox transgenic lines (n = 8). Period estimates vs R.A.E for leaf movement in LL or individual leaves Col-0 and *AtCCA1*-ox (*AtCCA1*-ox 038) (c) and Ws-2 and two independent transgenic lines of *HvCAA1-ox* (d). *n* = 30. All experiments were independently repeated at least twice. Ws-2 (closed squares), Col-0 (closed triangles) *AtCCA1*-ox (open triangles) and two independent *HvCCA1-*ox transgenic lines (8–3 and 18–1) (open squares and diamonds).

Over-expression of *AtCCA1* in Arabidopsis abolishes circadian rhythms in multiple outputs of the circadian clock [25]. Here we investigated whether *HvCCA1* is functionally orthologous to *AtCCA1* by assaying circadian regulation of leaf movement, delayed chlorophyll fluorescence, transcript abundance and gas exchange in Arabidopsis overexpressing *HvCCA1* driven by 2x 35S cauliflower mosaic virus promoters (*HvCCA1-*ox; (S1 Fig). In continuous light (LL), Ws-2 and Col-0 wildtypes had robust circadian rhythms of delayed fluorescence (Ws-2 = 23.4 h; Col-0 = 23.6 h; Table 1, Fig 1) and leaf movement (Ws-2 = 24.1 h; Col-0 = 23.6 h; Table 1, Fig 1). By contrast, in *AtCCA1-*ox [25] and *HvCCA1*-ox lines circadian rhythms of both delayed fluorescence and leaf movement were severely disrupted (Fig 1B, 1C and 1D) and there was a lower percentage of rhythmic plants (defined as RAE > 0.5; Table 1). Similarly, rhythms of stomatal conductance and photosynthesis were abolished by *HvCCA1*-ox and *AtCCA1*-ox (Table 2). Of seven *HvCCA1-*ox plants, only one plant had detectable rhythms of both CO_2_ assimilation (34.5 h, 0.4 RAE) and transpiration (24.4 h, 0.3 RAE). One other plant had rhythmic CO_2_ assimilation in LL (22.9 h, 0.5 RAE). In contrast, leaf gas exchange of the Ws-2 wildtype background line had robust circadian rhythms. Of eight Ws-2 plants, five had circadian rhythms of CO_2_ assimilation (23.7 ± 0.3 h, 0.3 ± 0.1 RAE) and four Ws-2 plants, had detectable circadian rhythms of transpiration (23.2 ± 0.3 h, 0.3 ± 0.1 RAE).

**Table 1:** Summary of mean circadian period estimates, standard error (SE) and percent rhythmic (%Rh) for leaf movement (LM) and delayed fluorescence (DF).

	LM data				DF Data			
Line	Period (h)	SE	n	%Rh	Period (h)	SE	n	%Rh
Col	23.6	0.1	30	93	23.6	0.1	8	100
*CCA1-ox*	26.6	1.0	30	70	24.9	0.9	8	38
WS	24.1	0.1	30	100	23.4	0.1	8	100
8–3	20.0	0.6	30	83	25.7	0.6	8	75
14–2	20.4	1.1	30	80	N/A	N/A	N/A	N/A
16–4	24.2	0.1	30	87	27.8	1.6	8	75
17–7	24.1	0.2	30	87	21.2	1.1	8	38
18–1	24.2	0.3	30	77	25.9	1.7	8	75
19–4	24.8	0.1	30	90	24.7	1.3	8	75

N/A–no data available. Col indicates Col-0 and WS indicates Ws-2 ecotypes.

**Table 2:** The effect of *HvCCA1*-ox on circadian rhythms of photosynthesis, transpiration rate and stomatal conductance in Arabidopsis.

	Photosynthesis				Transpiration rate				Stomatal conductance			
Line	Period (h)	SE	n	%Rh	Period (h)	SE	n	%Rh	Period (h)	SE	n	%Rh
Col	23.5	0.4	5	100	24.6	0.5	5	60	24.7	2.0	5	80
*CCA1-ox*	26.4	8.5	5	45	26	2.2	5	60	29.2	0.4	5	60
WS	23.4	0.4	8	75	23.5	0.2	8	86	23.5	0.3	8	86
8–3	28.7	5.8	5	40	26.0	1.6	5	40	-	-	5	20
17.7	-	-	2	0	-	-	2	0	-	-	2	50

%Rh–percentage of rhythmic plants. “–” no rhythm could be assigned during data analysis. Col indicates Col-0 and WS indicates Ws-2 ecotypes.

**Fig 2:**
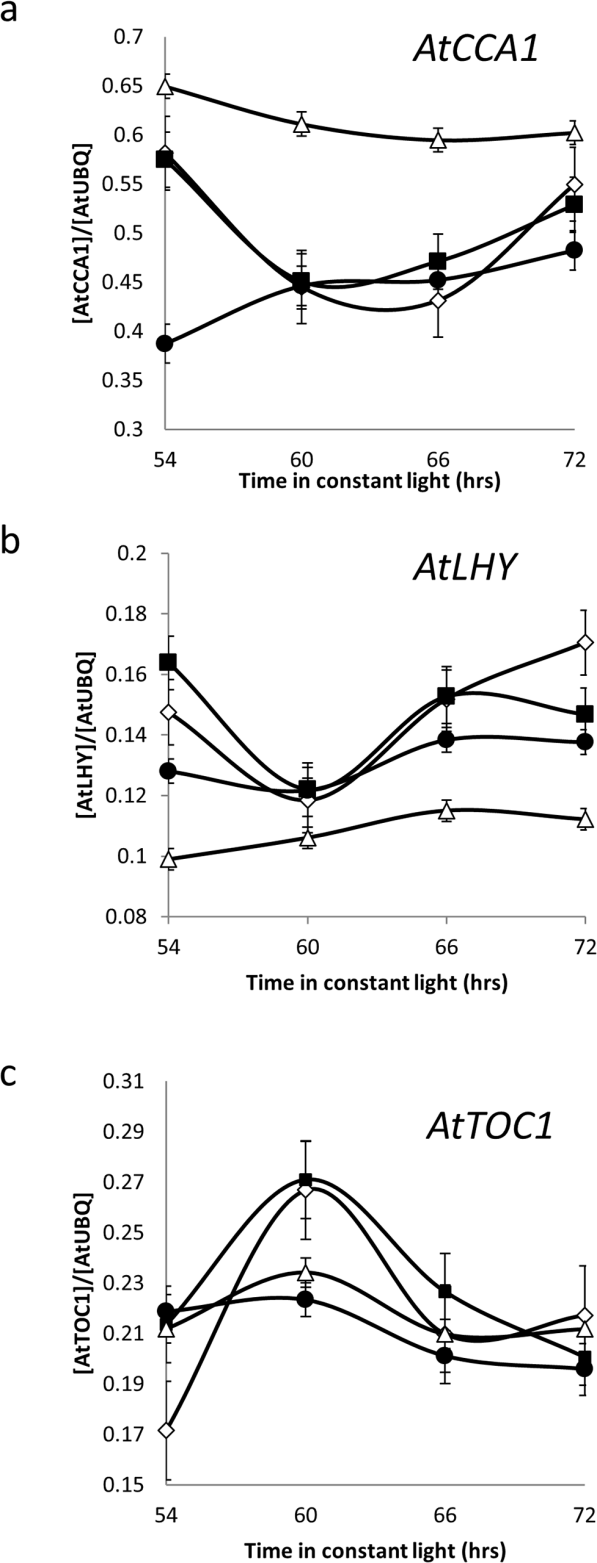
Overexpression of *HvCCA1* or *AtCCA1* (*AtCCA1*-ox 038) in Arabidopsis abolishes rhythms of transcript abundance of (a) *AtCCA1*, (b) *AtLHY* and (c) *AtTOC1*. *Ws-2* (white diamonds), *HvCCA1-ox* (line *8–3* (filled circles), *Col-*0 (black squares) and *AtCCA1*-ox 038 (white triangle). Transcript abundance was normalised to *UBIQUITIN10*.

Since analysis of circadian rhythms of delayed fluorescence, leaf movement, stomatal movements and photosynthesis all indicated that heterologous overexpression of barley *HvCCA1* made Arabidopsis plants circadian arrhythmic, we next investigated the effects of *HvCCA1*-ox on the expression of the circadian oscillator genes *AtCCA1*, *AtLHY* and *AtTOC1*. Over-expression of *HvCCA1* or *AtCCA1* resulted in arrhythmic or reduced amplitude expression of the endogenous *AtCCA1*, *AtTOC1* and *AtLHY* in LL (Fig 2).

### Overexpression of *HvCCA1* causes morphological and developmental defects in Arabidopsis

**Fig 3:**
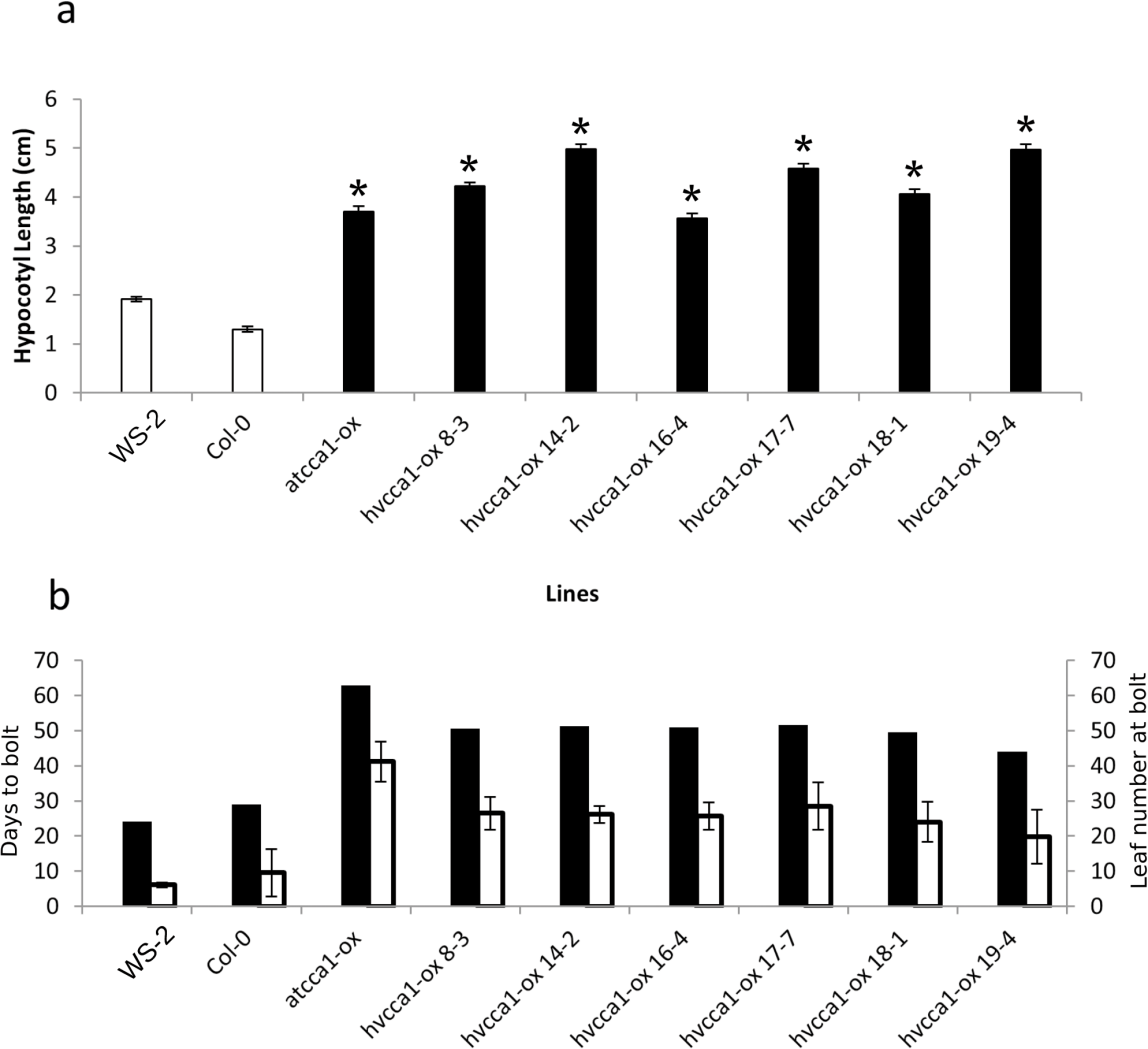
Over expression of *HvCCA1* in Arabidopsis causes elongated hypocotyls and delayed flowering. (a) Hypocotyl length was measured in WS and Col-0 background (white bars) or *AtCCA1*-ox (*AtCCA1*-ox 038) or *HvCCA1*-ox (indicated by the independent transgenic line numbers). Stars indicate significant difference in the hypocotyl experiments. (b) Bolting time in days (black bars) and numbers of leaves at bolting (white) in Ws-2 (WS) and Col-0 background or *AtCCA1*-ox (*CCA1*-ox) or *HvCCA1*-ox (indicated by the independent transgenic line numbers) when grown in 16h light, 8 h dark.

Rhythmic regulation by *AtCCA1* has profound effects on Arabidopsis development, such as the elongation of the hypocotyl, and the transition from vegetative to reproductive growth. We next investigated whether *HvCCA1* regulates development similar to *AtCCA1*. Over expression of both *AtCCA1* and *HvCCA1* resulted in longer hypocotyls, with those of *HvCCA1*-*ox* lines being significantly longer (3.5 to 5 mm) than the Col-0 and Ws-2 controls (1.5 mm and 2 mm; p<0.001; Fig 3A).

When grown under 16 h light 8 h dark, *AtCCA1*-ox delayed bolting and increased the number of leaves at bolt and this was phenocopied by over expression of *HvCCA1*. *HvCCA1-*ox lines flowered at least 20 days later than the wild type Ws-2 (p<0.001; Fig 3B) consistent with the *CCA1-*ox-038 line, which bolted approximately 30 days later than the control Col-0. The number of rosette leaves at bolting was also significantly higher in *HvCCA1*-ox plants compared to Ws-2 (p<0.001; Fig 3B).

### Analysis of the function of *HvPpd-H1* in barley

**Fig 4:**
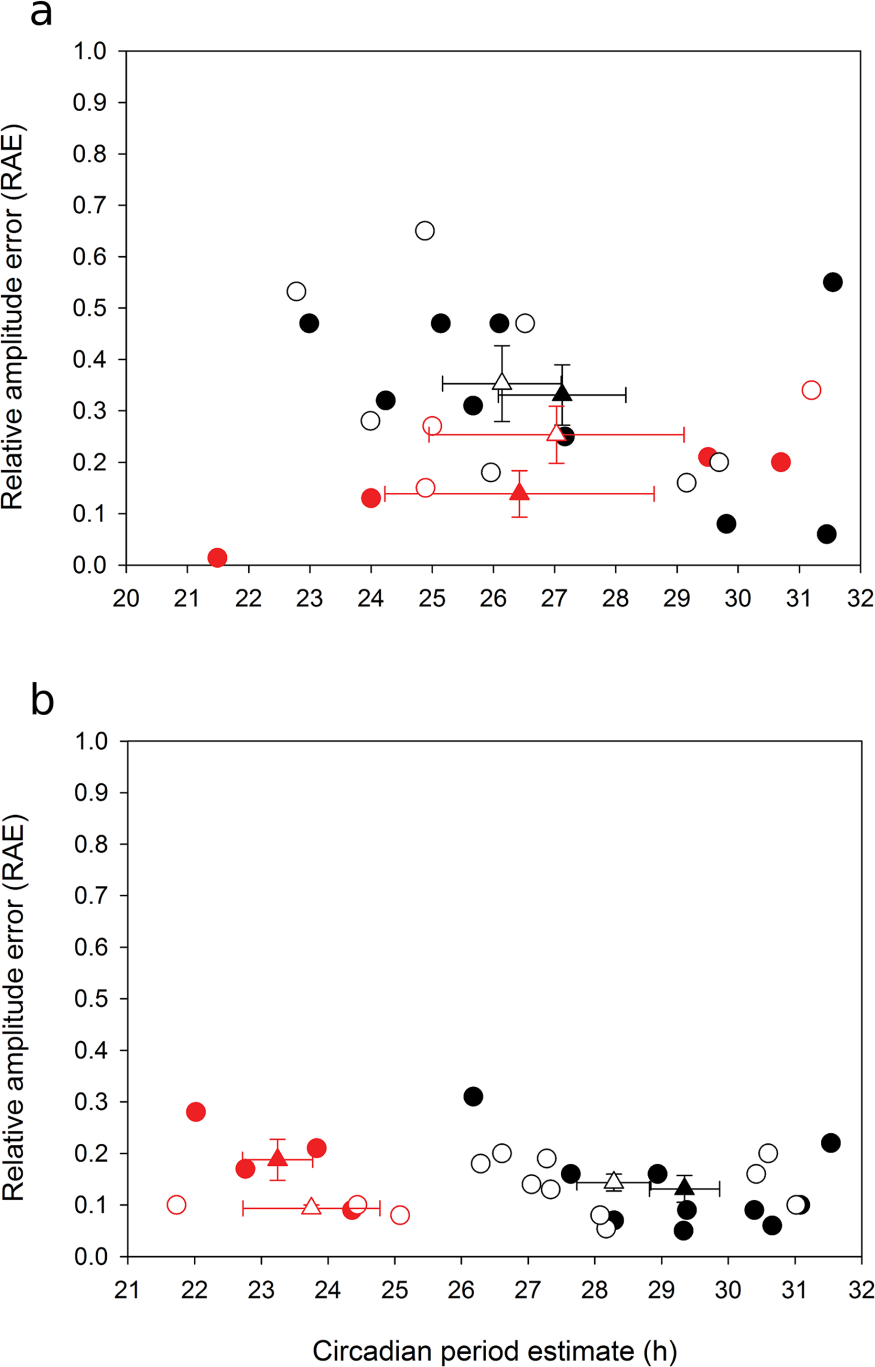
Estimates of period of circadian rhythms of (a) photosynthesis and (b) stomatal conductance in *HvPpd-H1* (filled circles) and *Hvppd-H1* (open circles) in constant white light of 100 μmol m^-2^ s^-1^ (black outlined symbols) or red light (red symbols). Each data point is derived from one individual seedling. Triangles represent the mean values.

We next investigated whether *HvPpd-H1* is similar in function to the Arabidopsis morning loop gene *AtPRR7*, with which *HvPpD-H1* shares most similarity. We analysed the function of the photoperiod responsive *HvPpd-H1* and photoperiod unresponsive *Hvppd-H1* variants to determine whether this locus can function in circadian regulation and whether natural mutations that alter photoperiodic sensitivity can affect circadian function. Circadian rhythms of stomatal conductance and CO_2_ assimilation were measured in *Ppd-H1* and *ppd-H1 H*. *vulgare* plants to determine if the natural mutant form *ppd-H1* had a consequence for circadian rhythms in barley. No significant difference between *Ppd-H1* and *ppd-H1* in the period of circadian rhythms of CO_2_ assimilation and stomatal conductance was detected (Fig 4). Measurement of gas exchange in barley is a low throughput assay in which only one individual at a time can be recorded. We obtained 15 measurements in white light representing >75 days of continuous measurement. To ensure that the assay, despite its low-throughput nature had sufficient sensitivity to report changes in circadian period, we also investigated the effects of red light on circadian period. As expected the circadian period in equal intensity illumination was shorter under red than white light and this was most clear when assaying rhythms of stomatal conductance (Fig 4B). This demonstrated that whilst measurements of circadian rhythms of assimilation where not robust, the assay of stomatal conductance had sufficient sensitivity to detect period alteration when present (Fig 4A and 4B). The effect of both *Ppd-H1* and *ppd-H1* and also was monitored in red light, because it has previously been demonstrated that in Arabidopsis circadian period phenotypes in *prr7*-*11* were more pronounced in red, rather than white light [26]. However, even in red light there was no significant difference between the period of the rhythms in *Ppd-H1* and *ppd-H1* (Fig 4).

### *HvPpd-H1* complements *AtPRR7-*11

The lack of a detectable effect of *Hvppd-H1* on circadian rhythms in barley might suggest that the mutation which regulates photoperiodic sensitivity does not affect the function of the circadian clock. Additionally, this finding raises the possibility that *HvPpd-H1* is not a circadian clock gene in barley, despite the sequence similarity to *AtPPR7*. We proceeded to use heterologous expression of barley genes in Arabidopsis to test whether *HvPpd-H1* can function as a circadian clock gene. Arabidopsis Col-0 and the *Atprr7-11* loss of function mutant were transformed with *HvPpd-H1* and *Hvppd-H1* under the control of the *AtPRR7* promoter resulting in elevated expression of the transgenes (S2 Fig). We reasoned that complementation of the long circadian period phenotype of *prr7*-*11* by heterologous expression of *HvPpd-H1* would provide evidence for *HvPpd-H1* acting in the circadian clock of barley.

The mean period of leaf movement rhythms of *prr7-11* plants was significantly longer than that of the wildtype (*prr7-*11 26.6 ± 0.3 h compared to Col-0 24.9 ± 0.2 h P = 0.0046). The *prr7-11* mutation was complemented by one of two lines of *pAtPRR7*::*Ppd-H1* and both lines of *pAtPRR7*::*ppd-H1*, with the complemented lines not being significantly different from wild type, except in the case of line *prr7-*11-p*AtPRR7*:*Ppd-H1-*c (Table 3). Since three of the four lines complemented with natural variants of *HvPpd-H1* were restored to wild type circadian period, we conclude that *HvPpd-H1* in barley is likely to have a role orthologous to *AtPRR7* in Arabidopsis and further supports the finding of the gas exchange analyses that *Hvppd-H1* mutation is without effect on circadian rhythms.

**Table 3:** Summary of circadian period estimates for leaf movement in Col-0, *prr7*-11 and *prr7-*11 transformed with either *pPRR7*::*Ppd-H1* or *pPRR7*::*ppd-H1*.

Line	Period (h)	SEM	RAE	n	%Rh
Col-0	24.9	0.2	0.2	32	75
*prr7-*11*	26.6	0.3	0.2	29	59
*prr7*-11 *pAtPRR7*:*Ppd-H1-c*	25.8	0.3	0.2	35	69
*prr7*-11 *pAtPRR7*:*Ppd-H1-d* *	25.0	0.3	0.2	25	64
*prr7*-11 *pAtPRR7*:*ppd-H1-e* *	25.3	0.3	0.2	21	86
*prr7*-11 *pAtPRR7*:*ppd-H1-f* *	25.6	0.3	0.2	29	59

* indicates significant difference at 5% level compared to background.

The background for *prr7*-11 is Col-0. The background for the complemented lines is *prr7*-11. SEM = standard error of the mean. %Rh = Percentage of rhythmic seedlings.

## Discussion

We provide experimental evidence that *HvCCA1* and *HvPpd-H1* have functionality similar to *AtCCA1* and *AtPRR7* in Arabidopsis. Heterologous expression in Arabidopsis of *HvCCA1-ox* results in circadian arrhythmia and morphological changes consistent with *HvCCA1* being functionally equivalent to *AtCCA1*. Our data show that constitutive expression of *HvCCA1* in Arabidopsis causes arrhythmia in leaf movement, delayed fluorescence and several circadian clock genes. It also causes significant hypocotyl elongation and late flowering in *HvCCA1* transformants. These results are consistent with *AtCCA1*-ox Arabidopsis data and provide a strong support that *CCA1* and its function are highly conserved throughout the plant kingdom. Expression of either *HvPpd-H1* or *Hvppd-H1* under control of the endogenous *AtPRR7* promoter partially restores the wildtype period of circadian leaf movement rhythms in an *AtPRR7* tDNA insertion mutant (*prr7-11*) suggesting that the function of *Hv-Ppd1* is as a circadian clock gene. A similar experiment in rice provided evidence that the *OsPRR37* and *AtPRR7* genes are partially interchangeable [27]. Together these findings show that the clock-associated functions of *CCA1* and *PRR7* are conserved between Arabidopsis, rice and barley.

Since both the photoperiodic-sensitive and–insensitive alleles of *Hv-Ppd1* were able to complement the *prr7-11* phenotype, this suggested that the causative SNP affects photoperiodism independent of circadian rhythms. To test this directly, circadian rhythms of gas exchange from a Triumph (*ppd-H1*) *H*. *vulgare* line were compared to those from the same line into which the Igri *Ppd-H1* allele had been introgressed and it was found that the circadian period was not significantly altered between barley plants carrying the *Hv-Ppd1* and *Hv-ppd1* alleles. Whilst mutation in *HvPpd-H1* did not produce detectable effects on circadian function either in barley or through complementation tests in Arabidopsis *Atprr*-11, the *Hvppd-H1* allele does abolish circadian rhythms of photoperiodic-response and vernalisation-response genes *HvCO1*, *HvCO2* and *Vrn-H1* [12]. These findings coupled with our demonstration that both *HvPpd1-H1* and *Hvppd-H1* can complement *AtPrr7-11* suggest that *HvPpd-H1* has at least two regulatory roles, one required for proper functioning of the circadian system, and one required for generation of rhythmic outputs, and it is the latter that is compromised in *Hvppd-H1*.

The analysis of circadian rhythms in barley and the other grain crops is of critical importance, since domestication has required breeders and early farmers to select for varieties with altered photoperiodic sensitivity and adaptations for cold tolerance. These are both traits that are regulated by the circadian clock [1, 28]. We have described the use of gas exchange and heterologous expression in Arabidopsis circadian mutants as new tools for the dissection of the barley circadian signalling network and determined that *HvPpd-H1* is functionally ortholgous to *AtPRR7*.

## Supporting Information

S1 Fig Transcript abundance of *AtCCA1* (black) and 5’UTR *AtCCA1* (endogenous) in *AtCCA1-ox* (white) and *HvCCA1* in *HvCCA1*-ox plants (grey). Seedlings were entrained for one week in 12 light/12hr dark cycles at 22°C then transferred to constant light. Samples were taken 3 h after dawn. Lines used were Ws-2, Col-0, *AtCCA1*-ox 038 and *HvCCA1*-ox (8–3 and 18–1). Transcript abundance was normalised to Ubiquitin10.
(PNG)

S2 Fig Transcript levels of *HvPpd-H1* and *Hvppd-H1* relative to *PP2a* expression in *prr7-11* Arabidopsis seedlings transformed with *HvPpd-H1* and *Hvppd-H1* under the control of the *AtPRR7* promoter (mean ± s.d.; *n* = 3). (PNG)

S1 Table Sequences of the primers used in the course of this study. Forward (F) and reverse orientation (R) primers.
(PDF)
