# Supplementary figures and images for: Barley Hv CIRCADIAN CLOCK ASSOCIATED 1 and Hv PHOTOPERIOD H1 Are Circadian Regulators That Can Affect Circadian Rhythms in Arabidopsis
Source: PLoS One. 2015 Jun 15;10(6):e0127449. doi: 10.1371/journal.pone.0127449 (PMC4468191; doi:10.1371/journal.pone.0127449)

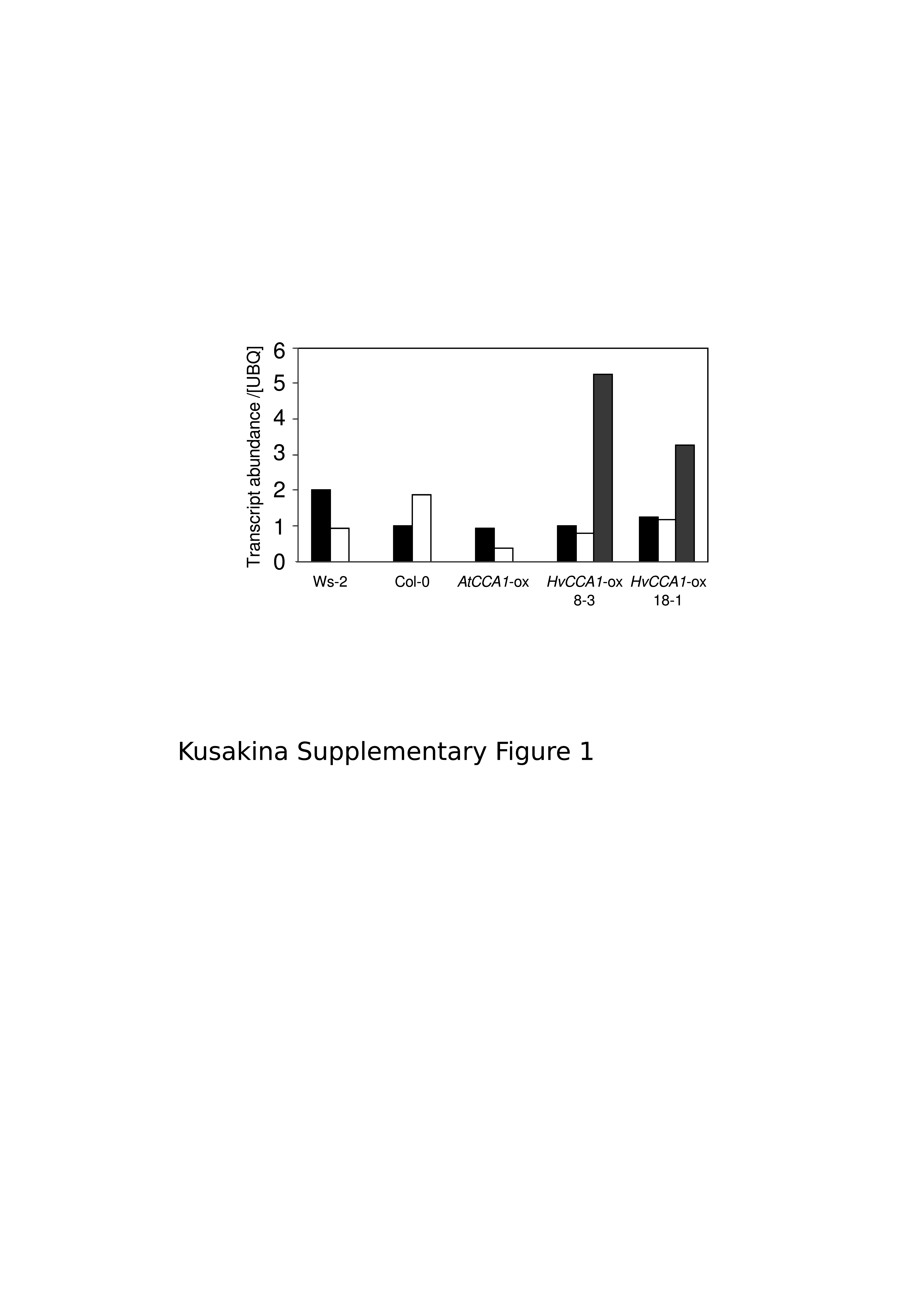

Supplement: S1 Fig — Seedlings were entrained for one week in 12 light/12hr dark cycles at 22°C then transferred to constant light. Samples were taken 3 h after dawn. Lines used were Ws-2, Col-0, AtCCA1-ox 038 and HvCCA1-ox (8–3 and 18–1). Transcript abundance was normalised to Ubiquitin10. (PNG) [file pone.0127449.s001.png]

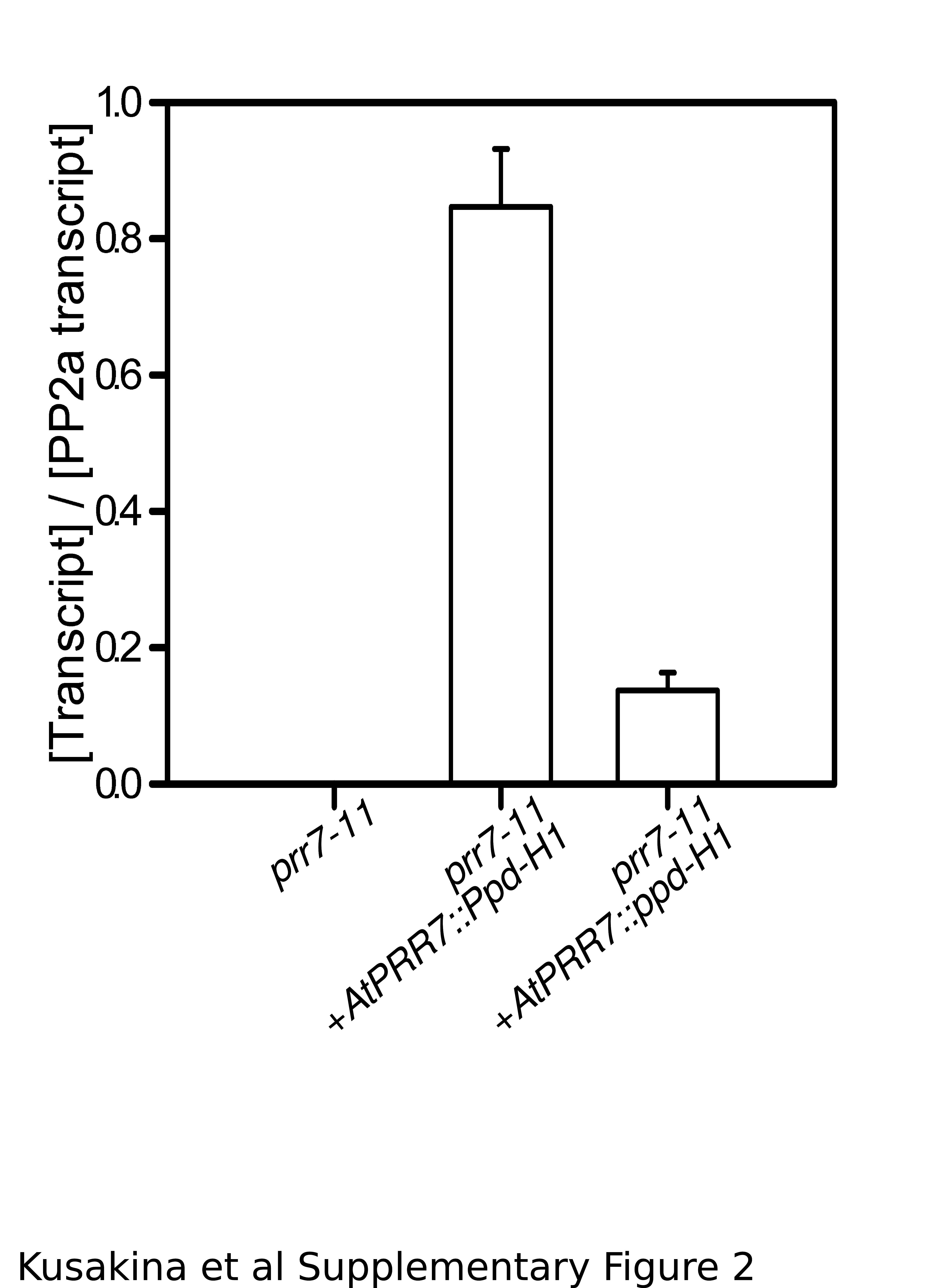

Supplement: S2 Fig — (PNG) [file pone.0127449.s002.png]
