# Supplementary material for: Barley Hv CIRCADIAN CLOCK ASSOCIATED 1 and Hv PHOTOPERIOD H1 Are Circadian Regulators That Can Affect Circadian Rhythms in Arabidopsis
Source: PLoS One. 2015 Jun 15;10(6):e0127449. doi: 10.1371/journal.pone.0127449 (PMC4468191; doi:10.1371/journal.pone.0127449)
Supplement: S1 Table — Forward (F) and reverse orientation (R) primers. (PDF) [file pone.0127449.s003.pdf]

Supplementary Table 1 Sequences of the primers used in the course of this study. Forward (F) and reverse orientation (R) primers.

Name	Primer sequence (5’ to 3’)
HvCCA1 – Forward	5’-CCTGGAATTGGAGATGGAGATAAATTCTTC-3’
HvCCA1 – Reverse	5’-GGAAGTAAATCACGTGGAGGGTTCGC-3’
CCA1 – Forward	5’-TCTGTGTCTGACGAGGGTCG-3’
CCA1 – Reverse	5’-ACTTTGCGGCAATACCTCTCTGG-3’
LHY – Forward	5’-CCTTCCGAACATTTCTTTGGT-3’
LHY – Reverse	5’-CAGAGACAAGAGACAAGACATGG-3’
UBQ – Forward	5’-GGCCTTGTATAATCCCTGATGAATAAG-3’
UBQ - Reverse	5’-AAAGAGATAACAGGAACGGAAACATAGT-3’
HvPpd-H1 Forward	5’-TTGAGCTGAGCCTGAAGAG-3’
HvPpd-H1 Reverse	5’- TATAGCTAGGTGCGTGGCG-3’
pAtPRR7 Forward	5’-GATACCGCGGTAACTACATCT-3’
pAtPRR7 Reverse	5’-GATACCGCGGTATCTCGAGAA-3’
